# Dynamics of Notch Pathway Expression during Mouse Testis Post-Natal Development and along the Spermatogenic Cycle

**DOI:** 10.1371/journal.pone.0072767

**Published:** 2013-08-28

**Authors:** Daniel Murta, Marta Batista, Elisabete Silva, Alexandre Trindade, Domingos Henrique, António Duarte, Luís Lopes-da-Costa

**Affiliations:** 1 Reproduction and Obstetrics, CIISA, Faculty of Veterinary Medicine, Technical University of Lisbon, Lisbon, Portugal; 2 Gulbenkian Institute of Science, Oeiras, Portugal; 3 Institute of Molecular Medicine, Faculty of Medicine, University of Lisbon, Lisbon, Portugal; Clermont Université, France

## Abstract

The transcription and expression patterns of Notch pathway components (Notch 1–3, Delta1 and 4, Jagged1) and effectors (Hes1, Hes2, Hes5 and Nrarp) were evaluated (through RT-PCR and IHC) in the mouse testis at key moments of post-natal development, and along the adult spermatogenic cycle. Notch pathway components and effectors are transcribed in the testis and expressed in germ, Sertoli and Leydig cells, and each Notch component shows a specific cell-type and time-window expression pattern. This expression at key testis developmental events prompt for a role of Notch signaling in pre-pubertal spermatogonia quiescence, onset of spermatogenesis, and regulation of the spermatogenic cycle.

## Introduction

Spermatogenesis, the generation of haploid, highly specialized germ cells (spermatozoa) in the testis, is the result of a complex orchestration initiated at puberty, involving continuous and serial cellular proliferation and differentiation events [1]. In the mouse, only Sertoli cells and spermatogonia are present in the seminiferous tubules until post-natal (pn) day 8, meiosis begins on pn day 10, round spermatids appear by pn day 18 after completing meiosis, and another 14 days are required for spermatids to complete their differentiation and to be released from the seminiferous epithelium [2], [3]. In this species, the spermatogenic cycle length is about 8.6 days and is divided in 12 stages arranged in order along the length of the seminiferous tubules [4], [5]. Leydig cells are interstitial endocrine cells that mainly secrete testosterone. In mammals, two main periods of Leydig cell function occur. A first Leydig cell generation develops during fetal life and the secreted testosterone is responsible for the masculinization of the urogenital system [6]. These cells regress thereafter, although some fetal Leydig cells persist in adult life [7]. A second Leydig cell population appears at puberty secreting testosterone required for the onset of spermatogenesis and overall maintenance of male reproductive function [6].

The gene pathways involved in the regulation of the highly complex cellular remodeling associated with spermatogenesis and male hormone secretion are poorly understood. Deciphering these mechanisms could potentially lead to the development of new therapeutic strategies addressed to male infertility and male contraception. The Notch pathway is an evolutionarily well-conserved system that has been implicated in cell fate decisions in several tissues [8], [9]. In mammals, four receptors (Notch1–4) and five ligands (three delta-like - Dll1, Dll3 and Dll4 - and two serrate-like - Jagged1 and Jagged2) were identified [9]. The Notch signaling pathway is activated by binding of the extracellular domain of the receptors with the ligands expressed on neighboring cells. This leads to the cleavage of the Notch intracellular domain by the γ-secretase complex and its translocation to the nucleus, where it associates with other transcriptional factors, thus regulating transcription of Notch target genes [9]. The hairy/enhancer of split (*Hes*) genes, coding for highly conserved proteins, are the more ubiquitous Notch effector genes [10]. In mammals, *Hes1* and *Hes5* are the more represented of these genes [9].

Notch signaling was implicated in germ cell development in *Caenorhabditis elegans* [11] and *Drosophila* [12]–[15], and components of the Notch pathway were reported to be present in the neonate and adult mammalian testis [16]–[24]. In rodents and humans, aberrant Notch activity was associated with male infertility [17], [19], [21], [22], [24], [25]. However, other studies [23], [26] reported that most of Notch pathway components are not transcribed in the mouse testis and that Notch blockage in germ and Sertoli cells had no effect on spermatogenesis. Therefore, the overall available information regarding the potential involvement of Notch signaling in spermatogenesis is fragmentary and controversial.

The objective of this work was to identify transcription and perform a detailed evaluation of the expression patterns of Notch pathway components during mouse testis pn development and, at adult life, along the spermatogenic cycle. Additionally, the transcription and expression of Notch effector genes in the adult testis was investigated to allow a conclusion regarding the activation of the pathway during spermatogenesis. Our main hypothesis is that Notch signaling might be involved in the regulation of spermatogenesis and male testis endocrine secretion. As a first approach, we identified transcripts in the adult testis by reverse transcription (RT)-PCR and then performed the immunolocalization (through immunohistochemistry - IHC) of Notch receptors and ligands in the testis at serial moments of the testis pn development (pn day 4; pn day 15; 1 month; 3 months). This was complemented with the identification of transcription of Notch effector genes and their immunolocalization (through IHC) in the testis during adult spermatogenesis.

Altogether, results strongly prompt for a role of Notch signaling in spermatogonia pool maintenance, onset of spermatogenesis, regulation of spermatogenesis pace, germ cells identity and differentiation, and Leydig cells function.

## Materials and Methods

### Animals

Experiments were conducted in compliance with the Portuguese legislation for the use of animals for experimental purposes (Decreto-Lei n° 129/92 and Portaria n° 1005/92, DR n° 245, série I-B, 4930-42) and with the European Union legislation (Directive n. 86/609/EEC, from the 24^th^ October 1996). Mice manipulation protocols were approved by the national regulatory agency (DGV – Direção Geral de Veterinária) and the Institutional Animal Care and Use Committee (CEBEA – Comissão de Ética e Bem-Estar Animal). All authors are accredited as FELASA category C scientists or equivalent.

CD1 mice were maintained in a 12-hour light/dark cycle, in ventilated cages with corn cob as bedding, and were given access to standard laboratory diet and water *ad libitum*. The mice health was routinely monitored. Outbred CD1 animals were chosen to introduce normal biological variability within the experiment.

### Experimental Design

Expression of Notch pathway components in the testis was evaluated at four time-points of pn development: pn day 4; pn day 15; 1 month; 3 months. CD1 male mice were euthanized through cervical dislocation under ketamine (15 mg/kg)/xylazine (1 mg/kg) anesthesia, followed by exsanguination. Four mice were allocated to each of the above four time-points and their testis were collected and processed for IHC Dissected testes from another four adult mice were individually processed for RT-PCR.

### Immunohistochemistry

Testes were fixed in 4% neutral phosphate buffered formalin at room temperature for 24 h and, after subsequent dehydration in ethanol, were embedded in paraffin. Spatial localization of expression of Notch pathway components (Notch1, Notch2, Notch3, Dll1, Dll4, Jagged1) and effectors (Hes1 and Hes5) was evaluated by IHC, according to a previously described method [27]. Slices were orientated transversally to the longitudinal axis of the testis, considering sequential sections of 3 µm and sequential twin-slides with each of the three cell markers for each Notch component (minimum of 3 slices per testis for each Notch component in all 16 animals). Tissue sections were stained by haematoxylin and identification of cell types was done through histology [28] and the use of cell markers in twin slides: The goat anti-3β-HSD antibody was used to identify Leydig cells and the rabbit anti-DAZL and anti-DDX4 antibodies were used to identify pre-meiotic and post-meiotic germ cells, respectively. The antigen retrieval step was performed in citrate buffer (10 mM, pH 6.0), except for both anti-Notch1, anti-Hes1, anti-Hes5 and anti-Jagged1 (ab7771) antibodies (Tris-EDTA, pH 9.0). Blocking was performed in PBS with 2% w/v bovine serum albumin (A7906, Sigma-Aldrich, Inc.) for one hour at room temperature. Tissue sections were incubated overnight at 4°C with the following primary antibodies: rabbit anti-Notch1 (Ab27526, Abcam, diluted 1∶100), rabbit anti-Notch2 (Ab8926, Abcam, diluted 1∶100), rabbit anti-Notch3 (Ab23426, Abcam, diluted 1∶160), rabbit anti-Dll1 (Ab10554, Abcam, diluted 1∶100), rabbit anti-Dll4 (Ab7280; Abcam, diluted 1∶200), rabbit anti-Jagged1 (SC-8303, Santacruz Biotechnology, diluted 1∶50), rabbit anti-Hes1 (Ab71559, Abcam, diluted 1∶100), rabbit anti-Hes5 (Ab25374, Abcam, diluted 1∶100), goat anti-3β-HSD (SC-30820, Santacruz Biotechnology, diluted 1∶300), rabbit anti-DAZL (Ab34139, Abcam, diluted 1∶250), and rabbit anti-DDX4 (Ab13840, Abcam, diluted 1∶200). The negative controls were performed with the polyclonal rabbit IgG (Ab27478, Abcam, diluted 1∶100) and, for the 3β-HSD antibody, with the goat control IgG (Ab37373, Abcam, diluted 1∶300). All primary antibodies were diluted in blocking solution. The peroxidase conjugated monoclonal mouse anti-goat/sheep IgG antibody (A9452, Sigma-Aldrich, Inc., diluted 1∶100) and the peroxidase conjugated goat anti-rabbit IgG polyclonal antibody (Dako 410972, diluted 1∶100) were used as secondary antibodies respectively for the anti-3β-HSD antibody, and the remaining primary antibodies. Staining was evaluated in the entire testis slice, considering several seminiferous tubules per slide. Expression patterns were established following the evaluation of a minimum of 24 slices (3 slices/testis×2 testis×4 animals) for each Notch component (plus 24 twin-cell marker slides) in each of the four time-points evaluated. In the case of Hes1 and Hes5, only nuclear staining was considered positive, indicating activation of Notch signaling. The antibodies for the Notch components and effectors were previously validated by others in the mouse (anti-Notch1 [29], anti-Notch3 [30], anti-Dll1, [31] anti-Dll4, [31] anti-Jagged1, [32] anti-Hes1 [33] and anti-Hes5 [34]) and rat species (anti-Notch2 [35]). Except for Notch1, all antibodies were polyclonal and the different lots used were originated from different animals (according to manufacturers). To further confirm the specificity of staining, the expression pattern obtained for Notch1 and Jagged1 was evaluated with different antibodies (the anti-Notch1, Ab52627, diluted 1∶50 and the anti-Jagged1, Ab7771, diluted 1∶100). We selected Notch1 (because all assays were performed with the same antibody lot) and Jagged1 (due to the specific expression pattern) as the targets for these repeats. Evaluation was performed on tissue sections from the same paraffin blocks of the same previously used animals. The two different antibodies against Notch1 and Jagged1 were simultaneously compared in twin slides (Figure S1).

### RT-PCR

Dissected testes of four additional CD1 male mice were individually collected, immediately frozen in liquid nitrogen and stored at −80°C until assay. RNA extraction, cDNA synthesis and mRNA transcription was performed as previously described [36]. The detection of Notch pathway genes (*Notch1, Notch2, Notch3, Dll1, Dll4, Jagged1*) and gene effectors (*Hes1, Hes2, Hes5, Nrarp*) were analyzed with specific primer pair sequences. Transcription of gene *HPRT1* was used as an endogenous control. Primer pair sequences are available upon request.

### Statistical Analysis

The relative frequency of pachytene spermatocytes expressing Notch2, Notch3 and Dll4 were determined by counting all marked and unmarked pachytene spermatocytes, in five different seminiferous tubules sections, at each of the spermatogenic cycle stages (I–II; III–IV; V–VI; VII–VIII; IX–X), in all testis. This totalized a mean of 1.005 (range: 812–1172) counted cells per animal. Differences between spermatogenic cycle stages were evaluated through ANOVA. As the Levene test for homogeneity of variances showed unequal variances between stages the Post Hoc test Tamhane’s T2 was performed. Significance was assumed at the 0.05 level (p<0.05).

## Results

### Notch Components and Notch Effectors are Transcribed in Adult Mouse Testis

**Figure 1:**
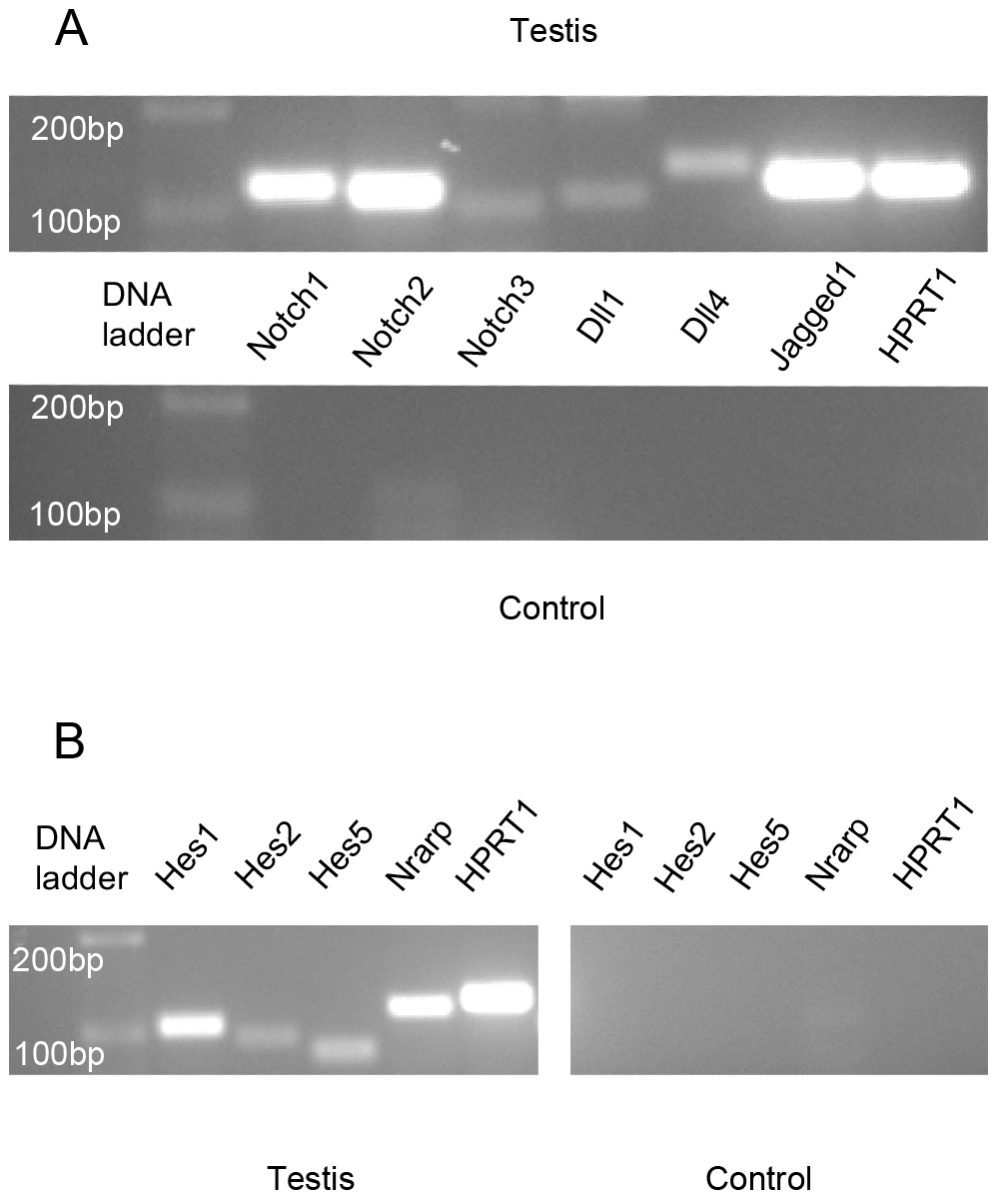
Transcription of Notch pathway components and effectors in the adult mouse testis, evidenced by RT-PCR. Gel electrophoresis RT-PCR results of: (A) Notch pathway gene transcripts - *Notch1, Notch2, Notch3, Dll1, Dll4, Jagged1* and *HPRT1* (endogenous control gene); (B) Notch pathway gene effectors - *Hes1, Hes2, Hes5, Nrarp* and *HPRT1* (endogenous control gene).

As shown in Figure 1, all the analyzed Notch pathway components and Notch pathway effectors are transcribed in the adult mouse testis, as evidenced by RT-PCR.

### Notch3 and Dll4 are Expressed in Spermatogonia and Sertoli Cells in pn Day 4 Mice Testis

**Figure 2:**
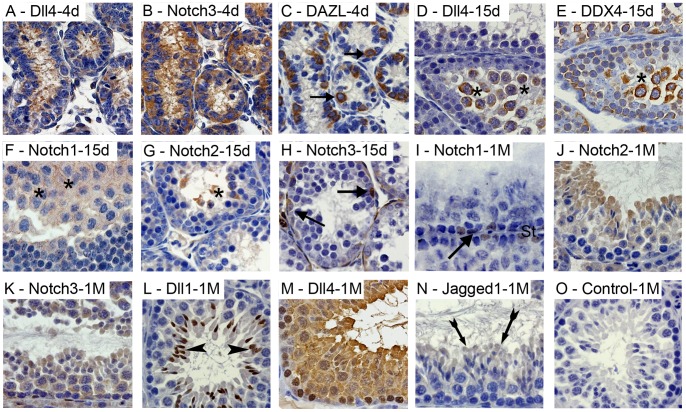
Immunolocalization of Notch pathway components in the post-natal (pn) developing testis. Positive immunostaining in brown color, counterstaining with haematoxylin (400×magnification). At pn day 4 (4 d), Dll4 (A) and Notch3 (B) are expressed in spermatogonia and Sertoli cells. Spermatogonia cells are marked with anti-DAZL antibody (C). At pn day 15 (15 d), Dll4 is expressed in germ cells initiating meiosis (D), marked with anti-DDX4 antibody (E). Notch1 (F) and Notch2 (G) are also expressed in germ cells entering meiosis. Spermatogonia express Notch3 (H). At 1 month pn (1 M), spermatogenic cycle stage IX-X (I–O), Notch1 is expressed in undifferentiated germ cells and Sertoli cells (I), while Notch2 and Notch3 are ubiquitously expressed in germ cells (J and K). Dll1 is specifically found in elongated spermatids nucleus (L) and Jagged1 in the elongated spermatids cytoplasm (N), while Dll4 is ubiquitously expressed in germ cells and in some Sertoli cells (M). Controls were done with rabbit IgG (O). Arrows point to spermatogonia; Asterisks mark germ cells entering meiosis; Arrow heads point to elongated spermatids; Tailed arrows point to elongated spermatids’ cytoplasm; st - Sertoli cells.

At pn day 4, when seminiferous tubules only present spermatogonia and Sertoli cells, only Notch3 and Dll4 are expressed in these cells (Figure 2 A and B). Spermatogonia were identified with anti-DAZL antibody in twin slides (Figure 2 C).

### Notch1, Notch2 and Dll4 are Expressed in Cells Initiating Meiosis at Puberty, on pn Day 15

At pn day 15, Dll4 expression is no longer observed in spermatogonia and become specific of cells initiating meiosis, which were marked with anti-DDX4 antibody in twin slides (Figure 2 D and E). Notch1 and Notch2 are also expressed in germ cells initiating meiosis (Figure 2 F and G), while Notch3 expression continues to be present in spermatogonia (Figure 2 H) but not in Sertoli cells.

### Dll1 and Jagged1 are First Expressed When Spermatids Start to Elongate at 1 Month of Life

At 1 month of age, spermatids are about to complete their differentiation and to be released from the seminiferous epithelium. All analyzed Notch components are expressed in the seminiferous tubules (Figure 2 I–N). Notch1 is expressed in spermatogonia and Sertoli cells (Figure 2 I). Notch2, Notch3 and Dll4 (Figure 2 J, K and M, respectively) are expressed in almost all germ cells. The most interesting feature at this time-point is the specific expression of Dll1 (Figure 2 L) and Jagged1 (Figure 2 N) in the elongated spermatids.

### Notch Pathway Components have a Unique Expression Pattern along the Spermatogenic Cycle in Adult Life

**Figure 3:**
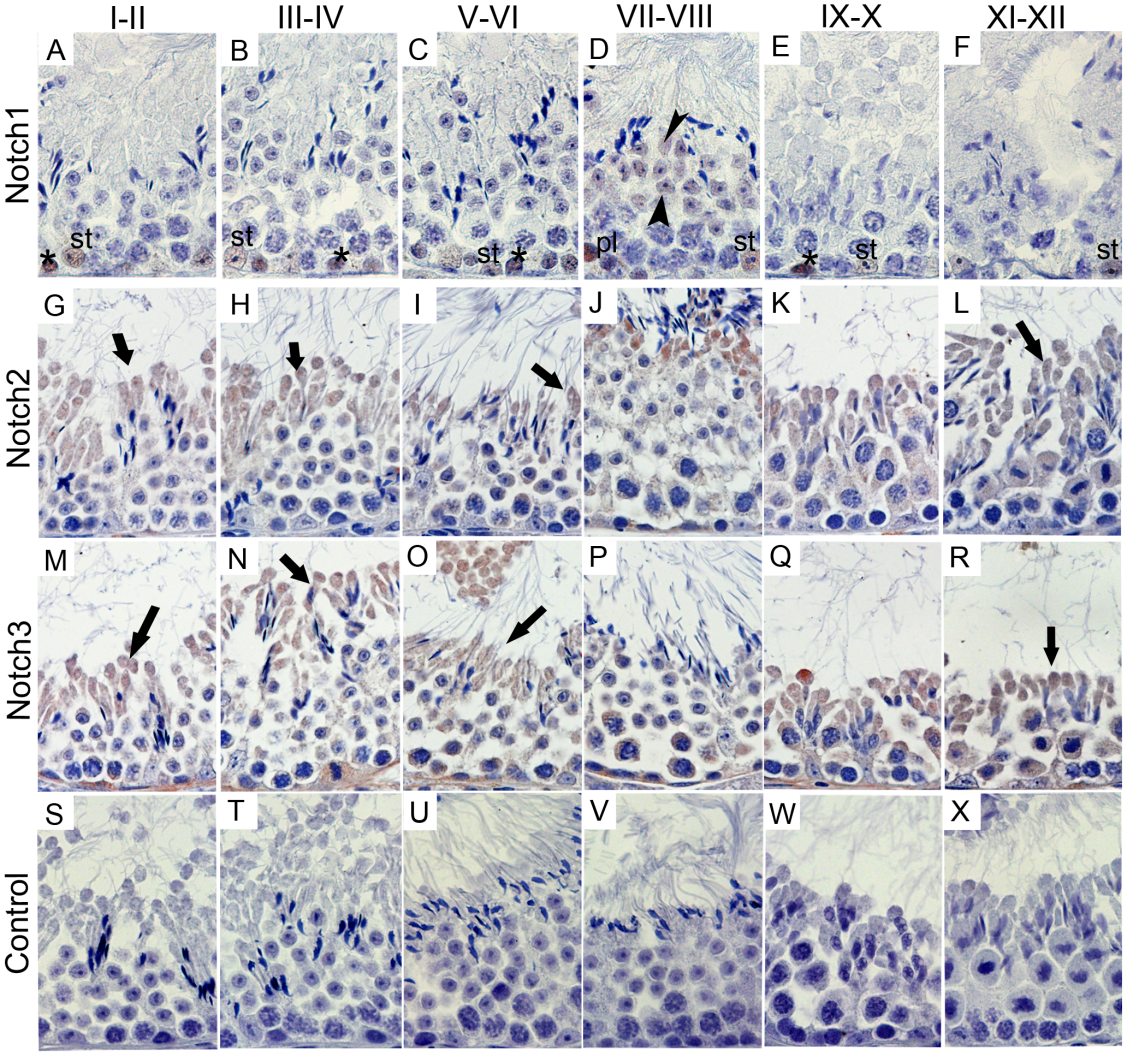
Expression of Notch pathway receptors during the spermatogenic cycle. Positive immunostaining in brown color, counterstaining with haematoxylin (400×magnification). Notch1 is expressed in spermatogonia and Sertoli cells at all spermatogenic stages (A–F), stage VII–VIII preleptotene spermatocytes and round spermatids (D). Notch2 is expressed in all germ cells, except spermatogonia and leptotene and zygotene spermatocytes (G–L). Notch3 is only absent from leptotene and zygotene spermatocytes (M–R). Notch2 and Notch3 are present in the elongated spermatid cytoplasm (G–R). Control was done with rabbit IgG (S–X). Asterisks mark spermatogonia; pl mark preleptotene spermatocytes; Arrow heads point to round spermatids; Arrows point to elongated spermatids’ cytoplasm; st - Sertoli cells.

**Figure 4:**
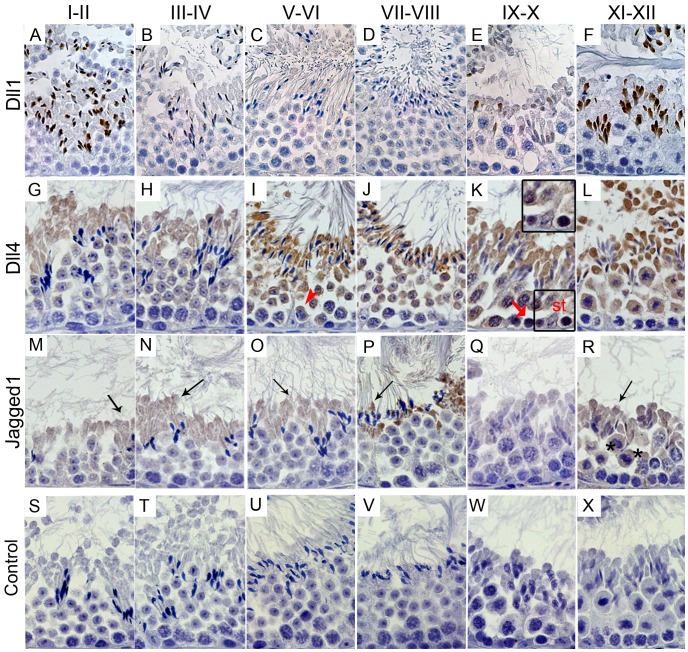
Expression of Notch pathway ligands during the spermatogenic cycle. Positive immunostaining in brown color, counterstaining with haematoxylin (400×magnification). Dll1 is specifically present in the head of elongated spermatids, between stage IX–X and stage III–IV (A–F). Dll4 show a dynamic expression pattern (G–L), being expressed mainly from stage V–VI pachytene spermatocytes onwards (I), but also in stage IX–X leptotene spermatocytes and Sertoli cells (K). Jagged1 is observed in elongated spermatids’ cytoplasm and in diplotene spermatocytes (M–R). Control was done with rabbit IgG (S–X). Arrow head point to stage V–VI pachytene spermatocytes; bold arrow point to stage IX–X leptotene spermatocytes; Arrows point to elongated spermatids’ cytoplasm; asterisks mark diplotene spermatocytes; st - Sertoli cells.

**Figure 5:**
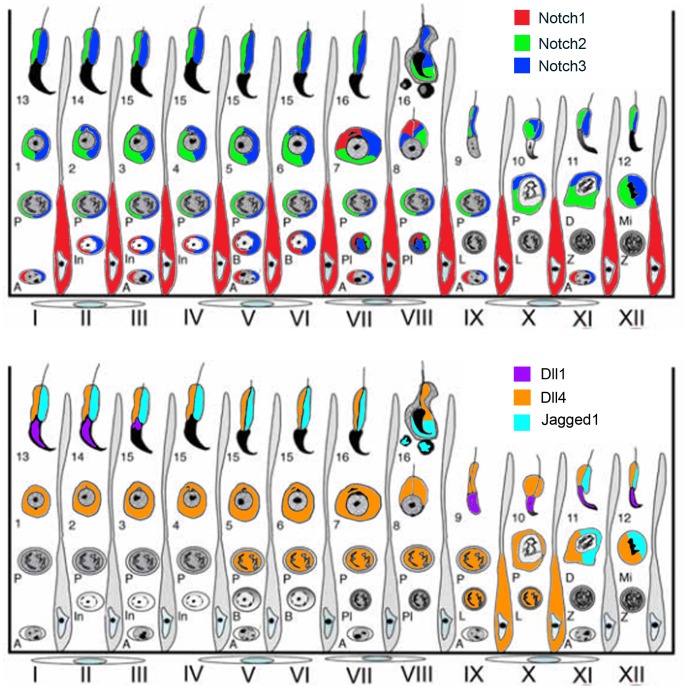
Schematic illustration of expression patterns of Notch pathway components along the spermatogenic cycle. Adapted draw-scheme [28] representing the mouse stages (I–XII) of the cycle of the seminiferous epithelium in the mouse. Cellular associations of layers of the seminiferous tubules, from the basement membrane to the lumen, are drawn with Sertoli cells separating each stage. Spermatogonia (A, In, B); spermatocytes (Pl- preleptotene, L- leptotene, Z- zygotene, P- pachytene, D- diakinesis, Mi- meiotic division); round spermatids (1–8); elongated spermatids (9–16). Localization of expression of Notch pathway components (top – receptors; bottom – ligands) is drawn in different colors, according to legend.

These expression patterns are illustrated in Figures 3 and 4, and resumed schematically in Figure 5. At adulthood (3 months of life), throughout the spermatogenic cycle, spermatogonia express Notch1 and Notch3 (Figure 3 A–C and M–O, respectively). When spermatogonia differentiate into preleptotene spermatocytes, at stage VII–VIII, these latter cells start to express Notch2 (Figure 3 J). Stage IX–X leptotene spermatocytes only express Dll4 (Figure 4 K). However, when leptotene spermatocytes differentiate into zygotene spermatocytes, at stage XI–XII, these latter cells cease to express the above Notch components.

**Figure 6:**
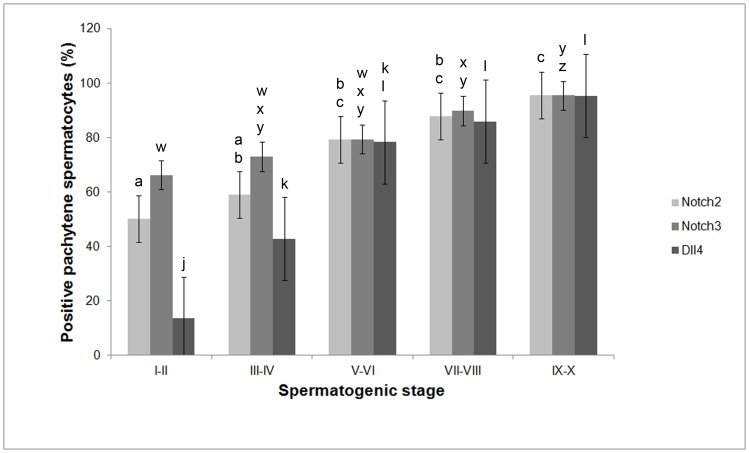
Evolution of the relative frequency of pachytene spermatocytes expressing Notch2, Notch3 or Dll4 along the spermatogenic cycle (stages I–II to IX–X). Error bars represent the standard error of the mean (SEM). Columns with different superscript differ significantly. Notch2: abc, *p*<0.05; Notch3: wxyz, *p*<0.05; Dll4: jkl, *p*<0.05.

From stage I–II to stage IX–X, pachytene spermatocytes increasingly express Notch2, Notch3 and Dll4 (Figure 3 G–K, M–Q and Figure 4 G–K, respectively). These data is graphically illustrated in Figure 6: from stage I–II to stage IX–X, the relative frequency of the cellular expression of Notch2, Notch3 and Dll4 significantly and gradually increases, respectively, from 50%, 66% and 14% to 95%. In the final step of meiosis, at stage XI–XII, diplotene spermatocytes also express Jagged1 (Figure 4 R). Following the second meiotic division and the formation of round spermatids, from stage I–II to stage V–VI, only Notch2, Notch3 and Dll4 expression is present (Figure 3 G–J, M–P and Figure 4 G–J, respectively). At stage VII–VIII, Notch1 is also expressed in round spermatids (Figure 3 D).

**Figure 7:**
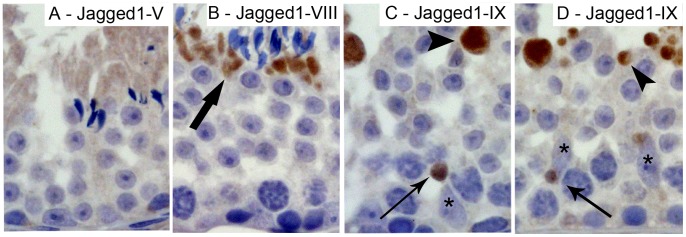
Jagged1 translocation within the residual bodies. Positive immunostaining in brown color, counterstaining with haematoxylin (400× magnification). Jagged1 is located in the elongated spermatids’ cytoplasm at stage V (A). At stage VIII, Jagged1 is located in the residual bodies at the sperm head tip (B). Following release of sperm cells from the seminiferous epithelium, at stage IX, residual bodies containing Jagged1 are progressively translocated towards the basement membrane and localized near the Sertoli cells nuclei (C–D). Bold arrow point to residual bodies containing Jagged1 in the sperm head tip. Arrow heads point to residual bodies containing Jagged1 at the luminal surface of the seminiferous epithelium. Arrows point to residual bodies containing Jagged1 near Sertoli cells nuclei. Asterisks mark Sertoli cells.

Dll1 starts to be expressed in the nuclear part of elongated spermatids when these cells begin to differentiate, at stage IX–X, and is expressed until stage III–IV (Figure 4 E, F, A and B). Notch2, Notch3 and Dll4 are also expressed in elongated spermatids at all stages but only in the cytoplasm (Figure 3 G–L, M–R and Figure 4 G–L, respectively). Jagged1 is located in the residual bodies of elongated spermatids between stage XI–XII and stage VII–VIII (Figure 4 R and M–P). This ligand seems to be translocated from the lumen to the basal region of the seminiferous tubules (Figure 7 A–D). This pattern is associated, following the release of sperm cells within the lumen of seminiferous tubules, with the engulfment of spermatids’ residual bodies by Sertoli cells and their transfer to the basement membrane region of the seminiferous tubule (Figure 7 C–D).

Both Notch1 and Dll4 are expressed in Sertoli cells. However, while Notch1 is present in Sertoli cells at all stages (Figure 3 A–F), Dll4 is only present in some of these cells at stage IX–X (Figure 4 K).

### Notch Pathway Effectors Hes1 and Hes5 are expressed during Adult Mice Spermatogenesis

**Figure 8:**
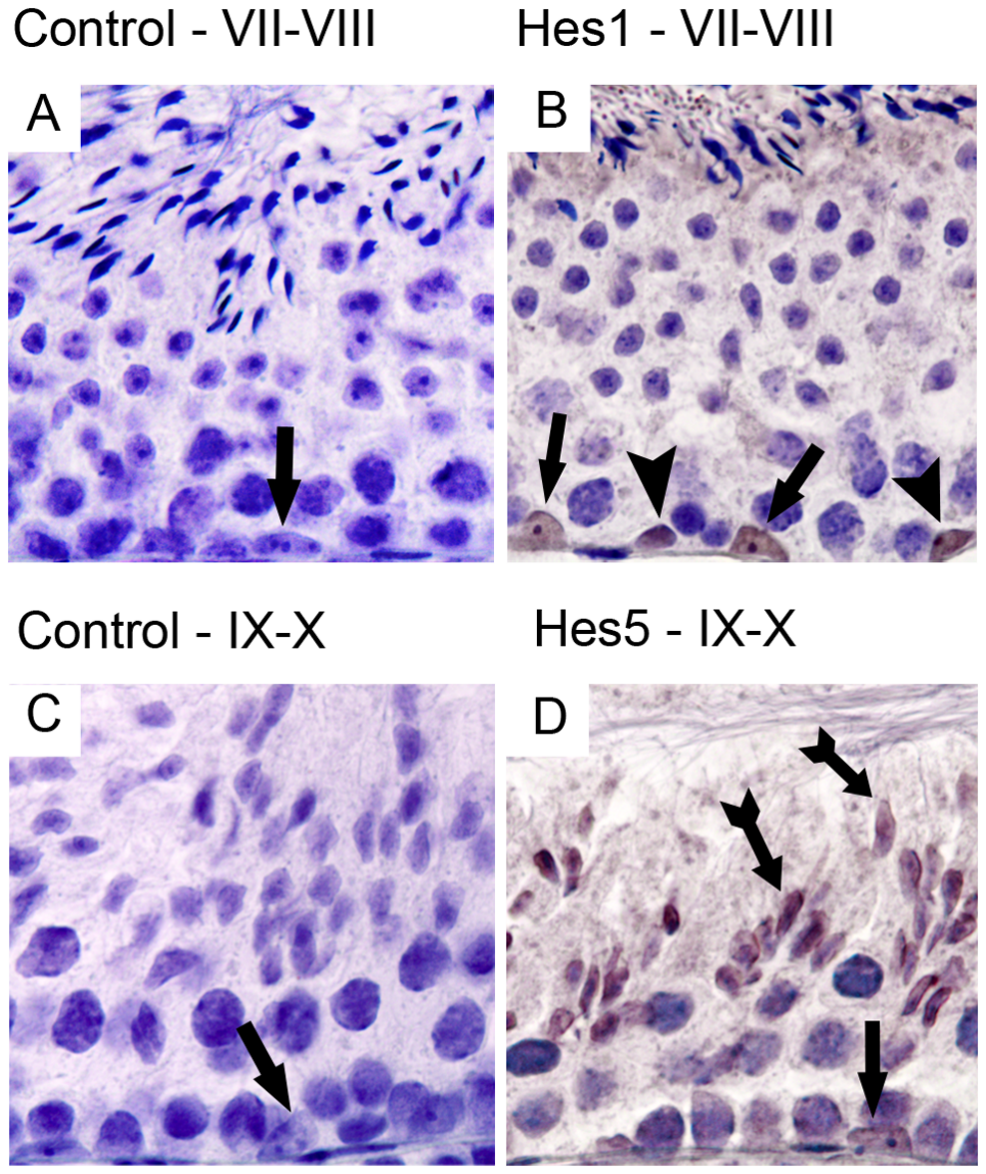
Expression of Notch pathway effectors Hes1 and Hes5 during adult spermatogenesis. Positive immunostaining in brown color, counterstaining with haematoxylin (400× magnification). Only nuclear staining was considered positive. Hes1 is expressed in stage VII–VIII Sertoli and spermatogonia cells (B). Hes5 is expressed in stage IX–X Sertoli cells and elongated spermatids (D). Control was done with rabbit IgG (A, C). Arrow heads point to spermatogonia cells. Arrows point to Sertoli cells. Tailed arrows point to elongated spermatids.

As shown in Figure 8, Hes1 is detected in Sertoli and spermatogonia cells, and Hes5 is detected in Sertoli cells and elongated spermatids.

### Notch Pathway Components Display a Dynamic Expression Pattern in Leydig Cells along Post-natal Testis Development

**Figure 9:**
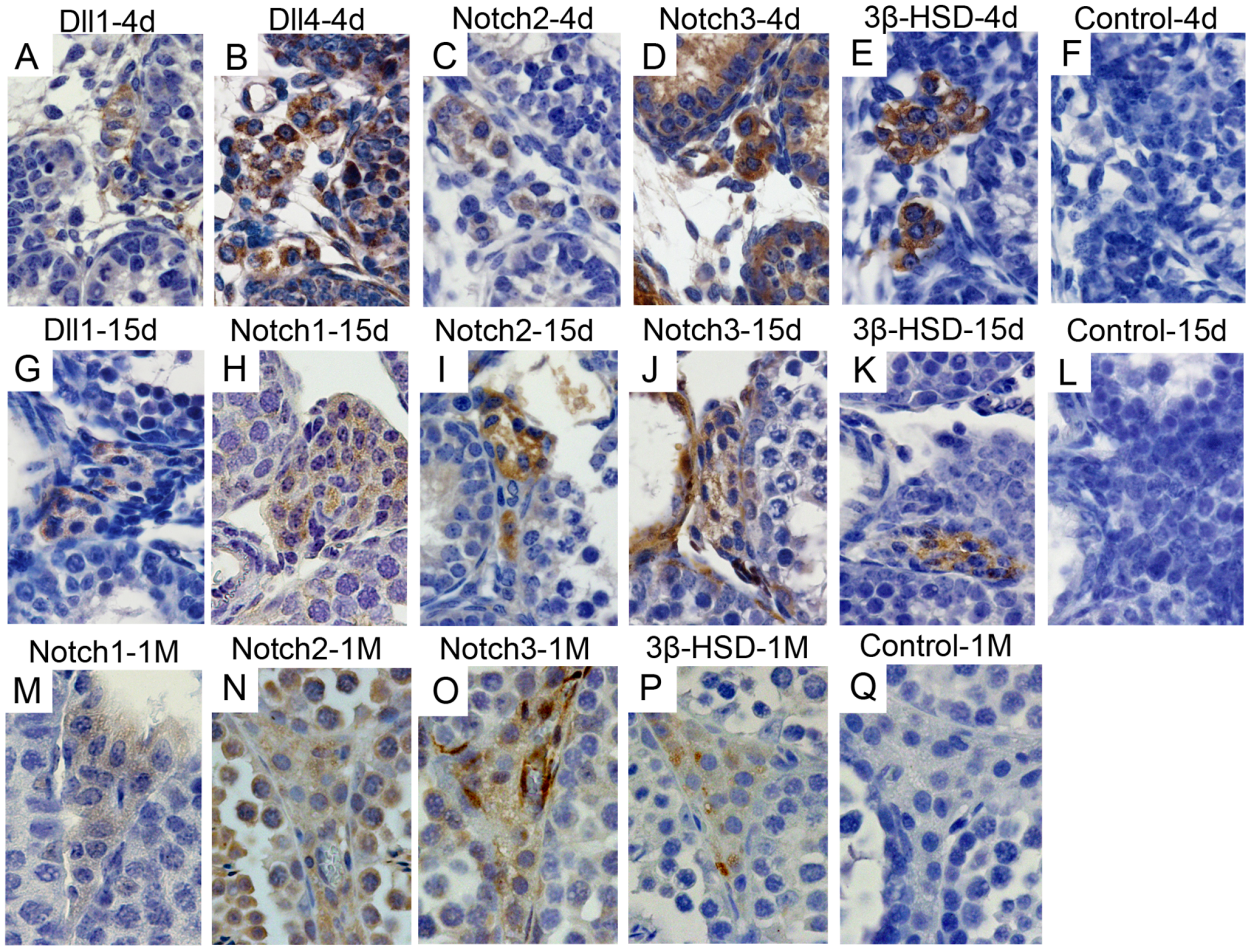
Dynamic expression of Notch pathway components in Leydig cells during mouse post-natal (pn) testis development. Positive immunostaining in brown color, counterstaining with haematoxylin (400× magnification). Expression of Dll1, Dll4, Notch2 and Notch3 is observed at pn day 4 (4 d) (A–D), while expression of Dll1, Notch1, Notch2 and Notch3 is present at pn day 15 (15 d) (G–J). Notch1, Notch2 and Notch3 continue to be expressed at 1 month pn (1 M) (M–O). Leydig cells were co-localized in twin slides with the anti-3β-HSD antibody (E,K,P). Control was done with rabbit IgG (F,L,Q).

**Figure 10:**
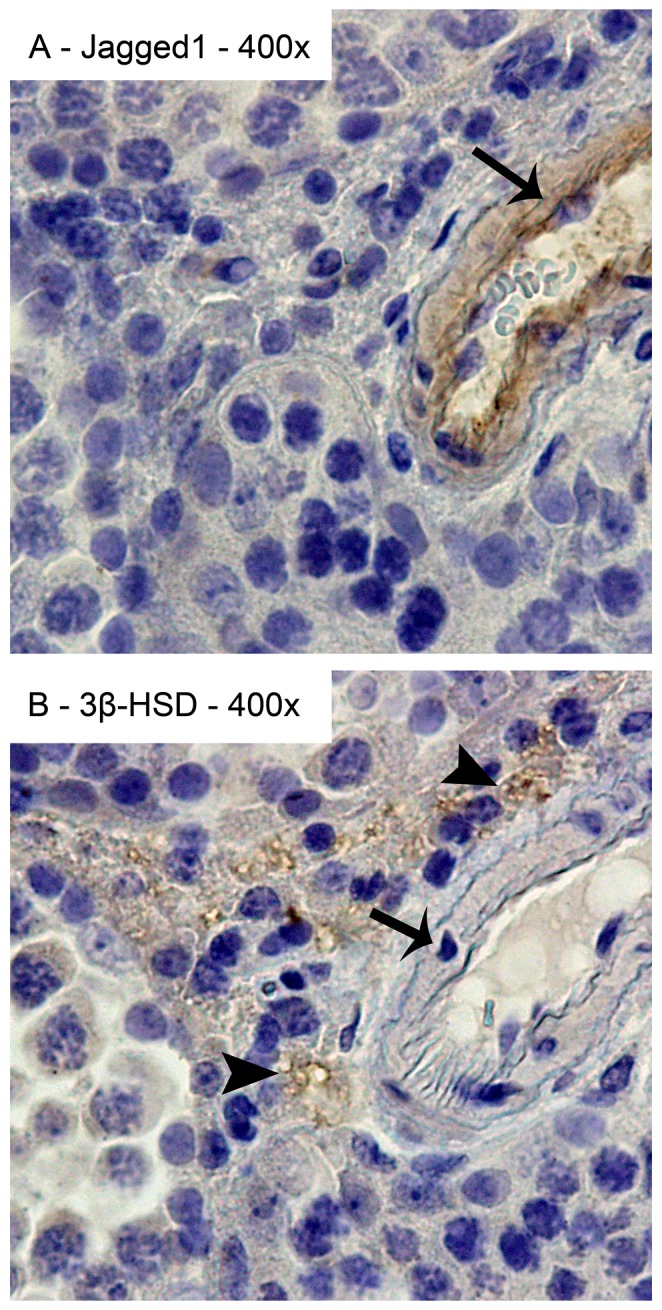
Jagged1 expression in endothelial cells of testis interstitial blood vessels. Positive immunostaining in brown color, counterstaining with haematoxylin (400x magnification). Jagged1 is present in endothelial cells of adult testis interstitial blood vessels (A). Leydig cells are in contact with the testis interstitial blood vessels. Leydig cells were co-localized in twin slides with the anti-3β-HSD antibody (B). Arrows point to endothelial cells. Arrow heads point to Leydig cells.

Leydig cells were identified in the interstitial space through the presence of 3β-HSD, an enzyme of the steroidogenic metabolic pathway (Figure 9 E,K,P). At pn day 4, Dll1, Dll4, Notch2 and Notch3 are expressed in these cells (Figure 9 A–D), whereas at pn day 15, expression of Dll1, Notch1, Notch2 and Notch3 is identified (Figure 9 G–J). From 1 month of life onwards only the Notch receptors continue to be expressed in Leydig cells (Figure 9 M–O). However, in adulthood, endothelial cells of blood vessels present in the interstitial space express Jagged1 (Figure 10).

## Discussion

Transcription of Notch pathway components is present in the adult mouse testis. The use of whole testis tissue for RT-PCR probably originates some background noise resulting from transcription outside the seminiferous tubules (endothelial cells, smooth muscle cells and mesenchymal cells) [37], [38]. Nevertheless, this result strongly leads to the suggestion that Notch pathway genes are transcribed in the seminiferous tubules during adult spermatogenesis. Furthermore, the observed transcription of Notch effector genes may indicate that Notch pathway is active during adult spermatogenesis.

Notch pathway components show a dynamic expression pattern in the mouse testis along the pn life. Although the expression of this pathway was already reported in the mammalian testis [16]–[24], this is the first integrated evaluation of expression of multiple Notch components in the testis along pn key time-points (pre-puberty, onset of puberty, adulthood). Additionally, our work is the first to describe Notch expression along the spermatogenic cycle. In pre-pubertal male mice, Dll4 and Notch3 are expressed in spermatogonia. As in the reported epithelial canonical function [8], [9], Notch signaling may be involved in maintaining the undifferentiated quiescent pool of spermatogonia. Dirami et al. [16] immunolocalized Notch 1–3, Dll1 (Dll4 was not screened), Jagged1 and Jagged2 in spermatogonia cells of pre-pubertal mice testis primary cell cultures, obtained as lysates. Inconsistencies between the above results and those obtained by us may arise from the use of different models. Disruption of testis architecture and in-vitro culture of testis cells may induce deviations in the gene expression profiles of cells. Hasegawa et al. [23], using an in-situ hybridization approach, only found transcripts of Jagged2 in spermatogonia of pre-pubertal mice. This inconsistency may result from a temporal dissociation between gene transcription and expression.

Dll4 and Notch3 are also expressed in Sertoli cells of pre-pubertal mice. These results contrast with those reported by Dirami et al. [16] using primary cell cultures (Notch2 and Jagged1 immunolocalized) and Hasegawa et al. [23] using an in-situ hybridization approach (Notch1 transcription detected). These inconsistencies may arise from comments addressed above for spermatogonia. Although it was reported that Notch signaling in Sertoli cells (and germ cells) has no effect on spermatogenesis [23], a recent study [24], also using genetically modified mice, showed that overexpression of Notch1 in Sertoli cells induces a depletion of the spermatogonia pool caused by an aberrant exit of gonocytes from the mitotic arrest. Therefore, as also prompted by our gene expression results, the above study [24] indicate a role of Notch signaling in the regulation of spermatogonia quiescence.

At onset of puberty Dll4 stops being expressed in spermatogonia and, together with receptors Notch1 and Notch2, undergoes expression in germ cells entering meiosis. Expression of Notch1 in germ cells entering meiosis was also described in rats [17]. These features prompt for a role of Notch signaling in the onset of meiosis, as also reported in *Caenorhabditis elegans* [39].

At adulthood, the specific dynamic expression of Notch pathway components in different cell types at different stages of the spermatogenic cycle suggests a central role of Notch signaling in the regulation of spermatogenesis. At stage VII–VIII of the spermatogenic cycle, when spermatogonia differentiate into pre-leptotene spermatocytes, all three Notch receptors are expressed, which may be related to spermatogonia fate, towards either self-renewal or differentiation. Expression of Notch1–3 in spermatogonia at adulthood was also reported by others [18], [20], [21], and the involvement of Notch in the regulation of spermatogonia fate was also suggested [40]. However, *Notch3* mutant mice were not infertile [41], suggesting that *Notch3* function is redundant in spermatogonia.

Stage IX–X leptotene spermatocytes only express the ligand Dll4. This ligand may be signaling back to the pre-leptotene spermatocytes, regulating their progression into the next differentiation step. Intriguingly, at stage XI–XII, when leptotene spermatocytes differentiate into zygotene spermatocytes, no expression of Notch pathway components is observed. The proportion of pachytene spermatocytes expressing Notch2, Notch3 and Dll4 increases from stage I–II to stage IX–X, when almost all these cells express these Notch pathway components. Here again, expression of Notch receptors 2 and 3 and the ligand Dll4 may be associated to cell differentiation progression. At stage XI–XII, at the final step of meiosis, Jagged1 is specifically expressed. Notch pathway expression was previously associated with cell division [42], [43]. Results here presented, prompt for a role of Jagged1 in the completion of meiosis.

We found all the three analyzed Notch receptors and Dll4 in round spermatids. Notch1 was only identified in stage VII–VIII, when these cells start to elongate. The expression of Notch receptors in round spermatids was already identified in mice [18] and rat [21]. Dll1 was only observed in the nuclear part of elongated spermatids. This cellular localization was unique among Notch ligands and receptors, as Notch2, Notch3, Dll4 and Jagged1 are also present in elongated spermatids, but in the cytoplasm. Residual bodies result from the elimination of part of the cytoplasm content of elongated spermatids, through an asymmetric cell partition [44], [45]. This asymmetric partition of cellular components may lead to changes in the distribution of Notch pathway components inside the cell, which may affect the ability of neighboring cells to deliver and receive Notch signaling.

Jagged1 is mainly expressed in the elongated spermatid cytoplasm. Interestingly, this ligand seems to be transported inside the residual body from the elongated spermatid to the adjacent Sertoli cell. Within Sertoli cells, this structure containing Jagged1 is transported from the luminal position of the seminiferous tubule to near the basement membrane. To the best knowledge of authors, this is the first report of such a Notch ligand translocation from one cell type to another. The effect of this engulfment is unknown. This could be associated with the activation of Notch1 in Sertoli cells or simply may represent a cell phagocytic function. The ectoplasmic specialization, the elongated spermatid anchoring system, maintains adherence between Sertoli cells and elongated spermatids, and also confers cell orientation and polarity within the seminiferous epithelium [46]. The Par complex proteins were associated to ectoplasmic specialization in spermatid orientation [46]. Notch signaling was associated to cell polarity decisions in tissues involving Par complex proteins, such as the neural epithelium [42], [47]. Here, the expression of Notch components near the elongated spermatid anchoring system may be associated with germ cell polarity definition and sperm release. Notch pathway was suggested to be associated with acrosome formation [17], [21]. Results here presented regarding Dll1 expression are consistent with a possible role in acrosome formation and overall sperm head shaping.

As RT-PCR detection of Notch effectors in whole testis does not discriminate between somatic and germ cells transcription, and since Notch signaling activation in germ cells remains controversial, we evaluated the activation of Notch pathway in adult spermatogenesis, through the nuclear localization of Notch effector proteins. From the Notch effector genes transcribed in the testis (see above), the more representative effector genes (*Hes1* and *Hes5*) were selected for this evaluation. The detection of these proteins (Hes1 and Hes5) in the nucleus indicates that the pathway is activated during adult spermatogenesis. Overall, the expression patterns identified in this study prompt for a role of Notch signaling in the regulation of spermatogenesis. This is supported by several other studies relating this pathway with male infertility [17], [19], [21], [24], [25]. In mice, treatment with specific antibodies directed against Notch1 and Jagged2 induced a spermatogenic blockage [17]. However, recently Hasegawa et al. [23] reported that Notch signaling was not required for normal spermatogenesis and Baptista et al. [26] reported that Notch1 expression was dispensable for spermatogenesis. These studies [23], [26] use genetic engineered mice with conditional deletions in *Pofut1* gene. Pofut1 protein is responsible for transfer of O-fucose to EGF repeats in Notch receptors, which alters receptor configuration [48]. However, in mammals, an unrelated endoplasmic reticulum α-glucosidase 1 can compensate for Pofut1 in promoting Notch folding and function and thus, Pofut1 is not absolutely required for stable cell surface expression of Notch [49]. Batista et al. [26] also evaluated mutant mice with conditional deletion of *Notch1* in spermatogonia cells and observed no phenotype in spermatogenesis and fertility. However, redundancy in Notch receptors function may be crucial to normal signaling since paralogues exert redundant or additive functions in maintaining the balance [50], [51]. Recently, Garcia et al. [24], using a GFP expression reporter driven by a RBP-J promoter, reported that Notch pathway is not active during spermatogenesis. The discrepancy between our results and those reported in the above study [24] is difficult to explain. In our study, the presence of the Notch pathway effectors (Hes1 and Hes5) in the nucleus was detected in the adult testis, both in Sertoli (as also reported in [23]) and germ cells which indicates that the pathway is active during spermatogenesis. The activation of Notch pathway during spermatogenesis was also proposed by others [17], [18], [21], [40].

Expression of Notch pathway components shows a dynamic pattern in Leydig cells along the pn life. Expression of Notch2, Notch3, Dll1 and Dll4 was observed in the pre-pubertal Leydig cells, while only the three Notch receptors (1–3) were observed in Leydig cells at adulthood. This change in the expression pattern may be related with the turn-over of the Leydig cell population (from embryonic to adult Leydig cells). During embryonic development, evolving blood vessels have a relevant role in testis morphogenesis, and expression of Jagged1 in interstitial cells was associated with maintenance of fetal Leydig progenitor cell populations [52]. In adult mice we observed expression of Jagged1 in endothelial cells of blood vessels surrounding Leydig cells. We hypothesize that a paracrine regulation of Leydig cell function may be in place through the signaling from neighboring endothelial cells, as reported for the endothelial-mesenchymal signaling [53].

## Conclusion

Here are reported the transcription and dynamic expression patterns of Notch pathway components along testis pn development and throughout the adult spermatogenic cycle. Results here described prompt for a role of Notch signaling in spermatogonia pool maintenance, onset of spermatogenesis, pace of the spermatogenic cycle, spermatid differentiation and regulation of pn Leydig cells function. We suggest that similarly to what happens in somitogenesis [54], [55], Notch pathway components may regulate the pace of spermatogenesis at several points, contributing to the coordination and orchestration of the complex proliferative and differentiation cellular events that take place along the spermatogenic cycle. This can be inferred from Figure 5 that schematically illustrates expression of Notch components along the spermatogenic cycle. As shown, specific combinations of receptor/ligand expression are associated with key events occurring during the spermatogenic cycle, namely differentiation of spermatogonia, onset and completion of meiosis, and spermatid differentiation. Notch signaling results from the specific activation of Notch receptors [56], [57]. In the complex cellular syncytium that constitutes the seminiferous epithelium, ligand expression by one cellular type may signal back to the preceding cell types, regulating their progression into the next differentiation step. Additionally, cell-autonomous Notch signaling may trigger forward cell differentiation. The presence of different receptors within a cell-type may be relevant for cell identity and function. The expression patterns observed address the need of future studies involving conditional specific Notch pathway mutant mice to evaluate the role of each Notch component in male reproductive function.

## Supporting Information

Figure S1 **Evaluation of the specificity of positive staining of Notch1 and Jagged1, using different antibodies.** Positive immunostaining in brown color, counterstaining with haematoxylin (400x magnification). Notch1: comparison between the anti-Notch1 (Ab27526) (A) and the anti-Notch1 (Ab52627) (B) antibodies; Positive staining was present in spermatogonia, round spermatids and Sertoli cells. Jagged1: comparison between the anti-Jagged1 (sc-8303) (C) and the anti-Jagged1 (Ab7771) (D) antibodies. Positive staining was present in the residual bodies at the sperm head tip. Control was done with rabbit IgG. Arrows point to spermatogonia cells. Asterisks mark Sertoli cells. Arrow heads point to residual bodies containing Jagged1 at the luminal surface of the seminiferous epithelium.
(TIF)
