# Supplementary figures and images for: Dynamics of Notch Pathway Expression during Mouse Testis Post-Natal Development and along the Spermatogenic Cycle
Source: PLoS One. 2013 Aug 28;8(8):e72767. doi: 10.1371/journal.pone.0072767 (PMC3755970; doi:10.1371/journal.pone.0072767)

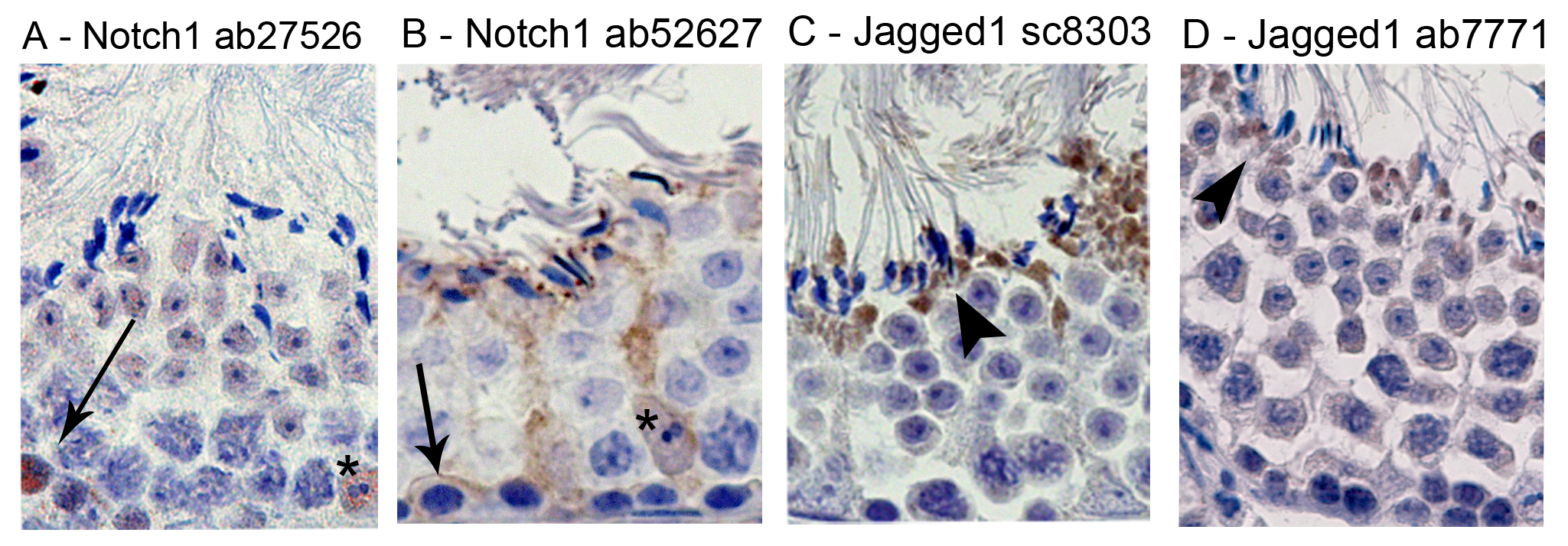

Supplement: Figure S1 — Evaluation of the specificity of positive staining of Notch1 and Jagged1, using different antibodies. Positive immunostaining in brown color, counterstaining with haematoxylin (400x magnification). Notch1: comparison between the anti-Notch1 (Ab27526) (A) and the anti-Notch1 (Ab52627) (B) antibodies; Positive staining was present in spermatogonia, round spermatids and Sertoli cells. Jagged1: comparison between the anti-Jagged1 (sc-8303) (C) and the anti-Jagged1 (Ab7771) (D) antibodies. Positive staining was present in the residual bodies at the sperm head tip. Control was done with rabbit IgG. Arrows point to spermatogonia cells. Asterisks mark Sertoli cells. Arrow heads point to residual bodies containing Jagged1 at the luminal surface of the seminiferous epithelium. (TIF) [file pone.0072767.s001.tif]
